# Higher baseline uric acid concentration is associated with non-attainment of optimal blood pressure

**DOI:** 10.1371/journal.pone.0236602

**Published:** 2020-07-27

**Authors:** Yuji Sato, Shouichi Fujimoto, Kunitoshi Iseki, Tsuneo Konta, Toshiki Moriyama, Kunihiro Yamagata, Kazuhiko Tsuruya, Ichiei Narita, Masahide Kondo, Masato Kasahara, Yugo Shibagaki, Koichi Asahi, Tsuyoshi Watanabe

**Affiliations:** 1 Division of Nephrology, Department of Internal Medicine, National Health Insurance Takachiho Town Hospital, Takachiho, Miyazaki, Japan; 2 Department of Hemovascular Medicine and Artificial Organs, Faculty of Medicine, University of Miyazaki, Miyazaki, Japan; 3 Steering Committee of Research on Design of the Comprehensive Health Care System for Chronic Kidney Disease (CKD) Based on the Individual Risk Assessment by Specific Health Check, Fukushima, Japan; International University of Health and Welfare, School of Medicine, JAPAN

## Abstract

A significant relationship exists between elevated uric acid concentration and both prevalent and incident hypertension; however, data regarding the influence of higher uric acid concentration at baseline on blood pressure control by antihypertensive drugs is scarce. Thus, a prospective cohort study was performed. The study outcome was the non-attainment of optimal blood pressure (NOBP). NOBP level was defined according to the Japanese hypertension guideline. This study enrolled a Japanese community-based cohort (N = 8,664; age 65.5 ± 6.4 years; women, 55.0%) who were not using antihypertensive drugs on the first visit for a health check-up program but started using antihypertensive drug(s) on the next-year visit. The participants were classified into quartiles based basic uric acid concentration. Odds ratios (ORs) were calculated for NOBP as the primary outcome measure. Multivariable logistic analysis showed that quartile 4 was significantly associated with NOBP when quartile 1 was set as the reference (OR (95% confidence interval), 1.36 (1.16–1.59), p<0.01), adjusted for potential confounders, such as age, sex, body mass index, presence of diabetes/dyslipidemia/chronic kidney disease (CKD), history of cardiovascular disease, daily drinking, and current smoking. In the subgroup analysis of female participants and participants with diabetes and CKD, a significant association was observed between +1 mg/dL of uric acid and NOBP. Higher uric acid concentration at baseline was significantly associated with NOBP on the first use of antihypertensive drug(s).

## Introduction

Studies reported significant relationships between higher uric acid level and prevalence of hypertension [1–3]. Moreover, some studies discussed whether higher uric acid was a cause or a phenomenon of hypertension. Recent basic research finding supports the fact that uric acid itself could cause hypertension. In essence, several studies reported about the significant relationship between higher uric acid level and incident hypertension [4–9]. In many of these studies, participants comprised the general population; however, one study demonstrated no significant relationship between higher uric acid level and incident hypertension in a type 2 diabetic cohort [9].

The basic mechanism underlying the cause of increased blood pressure (BP) by uric acid has been investigated; an animal model demonstrated an inhibition of nitric oxide generation of the vessels by uric acid, resulting endothelial cell damage [10], and uricase inhibitor-treated hyperuricemic rats showed intimal wall thickness of the afferent arterioles [11]. In hyperuricemia, the renin-angiotensin system in the kidney was activated [12], hyalinosis or narrowing of the arteriole was prominent in consecutive renal biopsy specimens [13], and the resistance index of the afferent artery was higher [14]. Further, a human study reported a positive correlation between serum uric acid level and salt sensitivity [15]. Wang et al attempted to explain how hyperuricemia causes incident hypertension by activated salt sensitivity [16]; hyperuricemia causes arteriolopathy, renal damage, and endothelial dysfunction via the activation of renin-angiotensin system, thus, resulting in the activation of salt sensitivity. In summary, higher uric acid can disrupt intrarenal hemodynamics by both functional- and anatomical mechanisms.

On the contrary, resistant hypertension (defined as an uncontrolled hypertension in spite of using three antihypertensive drugs) is a public health problem, and its prevalence has been reported to approximately range between 20 to 30% [17]. Certain risks contributing to resistant hypertension reported by a scientific statement from the “American Heart Association” [17] were as follows; older age, high baseline blood pressure, obesity, excessive dietary salt ingestion, chronic kidney disease (CKD), diabetes, left ventricular hypertrophy, black race, and female sex, among others; however, this statement did not refer to hyperuricemia. This study hypothesized that higher baseline uric acid level could be an additional factor that causes resistance to initial hypertensive therapy. To evaluate this hypothesis, we aimed to investigate the relationship between baseline uric acid concentration and whether or not optimal blood pressure is attained by patients from a large Japanese health check-up system who were prescribed antihypertensive medication for the first time.

## Materials and methods

### Study design and participants

This study was conducted as a part of the ongoing “Research on the Positioning of Chronic Kidney Disease in Specific Health Check and Guidance in Japan (SHCG)” project [18, 19]. Of the 47 prefectural governments in Japan, 27 agreed to participate in this study. SHCG data recorded between 2008 and 2010 were sent to and verified by the independent data center, the non-profit organization Japan Clinical Support Unit (Tokyo, Japan) [18, 19].

In this observational community-based cohort study, non-attainment of optimal blood pressure (NOBP) was set as the primary outcome measure. According to the Japanese hypertension guideline in 2004, optimal BP was defined as follows: systolic blood pressure (SBP) <140 mmHg and diastolic blood pressure (DBP) <90 mmHg as a basic principle, SBP <130 mmHg and DBP <85 mmHg in younger participants (aged <65 years), and SBP <130 mmHg and DBP <80 mmHg in participants with diabetes or CKD [20]. NOBP was defined as blood pressure which did not match the aforementioned optimal levels on second-year visit. The target subjects were Japanese citizens aged 40–74 years. Clinical and laboratory data were collected through this project from 2008 to 2010. To analyze the effect of antihypertensive drug use on BP, participants who did not use antihypertensive drugs on the first-year (2008 or 2009) visit but used antihypertensive drug on second-year (2009 or 2010) visit, were included.

**Fig 1:**
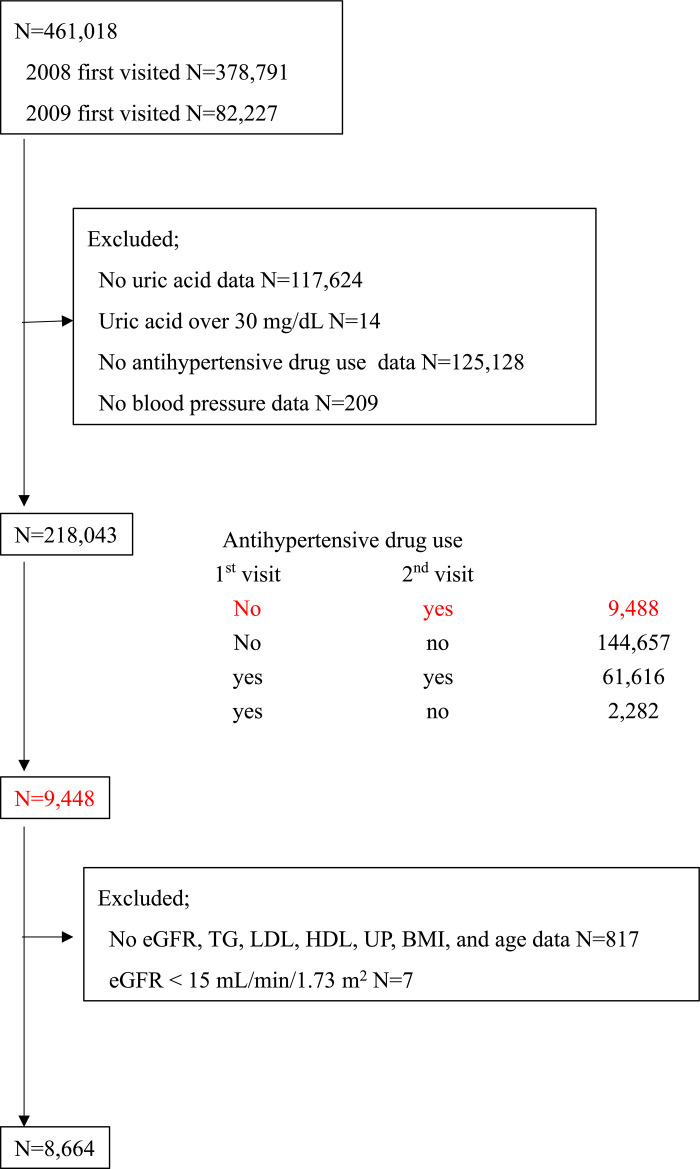
Participant enrollment. Of an initial sample size of 461,018 (378,791 and 82,227 participants who visited for the first time in 2008 and 2009, respectively), 242,975 participants were excluded because of missing uric acid data and unavailable information regarding antihypertensive drug (s) use, among others. Of 218,043 participants, 9,488 were eligible for this study (subjects who were not prescribed antihypertensive drugs on the first-year visit and prescribed antihypertensive drugs on the second-year visit). Further, 817 participants were excluded because of missing data on the following: eGFR, lipid, urine dipstick test, BMI, and age. In addition, seven participants were excluded because their eGFR was <15 mL/min/1.73 m^2^. Finally, 8,664 participants were enrolled in this study.

From an initial population of 461,018, which was the sum of 378,791 participants who visited for the first time in 2008 and 82,227 participants who visited health-checkup facilities for the first time in 2009, we excluded 242,975 participants because of the following reasons: missing baseline uric acid data (n = 117,624), uric acid level beyond 30 mg/dL (n = 14), no antihypertensive drug use data (n = 125,128), and no BP data (n = 209) (Fig 1). Many uric acid data were unavailable because uric acid measurement was not mandatory in this health check-up system. Among 218,043 participants, we identified 9,488 participants who were not prescribed antihypertensive drug(s) on the first year, but were prescribed antihypertensive drugs on the second year. Of these patients, 824 participants were excluded because of missing estimated glomerular filtration rate (eGFR), triglyceride (TG), low-density lipoprotein (LDL), high-density lipoprotein (HDL), dipstick urine protein, body mass index (BMI), and age data (n = 817), or had eGFR <15 mL/min/1.73 m^2^ (n = 7). Finally, 8,664 participants were enrolled in this study.

### Ethical consideration

This cohort study was conducted according to the guidelines of the Declaration of Helsinki and was granted ethics approval by the University of Miyazaki Review Board (IRB Approval Number # O-0117). Original Ethics Committee approval was obtained from Fukushima Medical University (IRB #1485, #2771). Similarly, this study was conducted according to the Ethical Guidelines for Medical and Health Research Involving Human Subjects enacted by MHLW of Japan [http://www.mhlw.go.jp/file/06-Seisakujouhou-10600000-Daijinkanboukouseikagakuka/0000069410.pdf and http://www.mhlw.go.jp/file/06-Seisakujouhou-10600000-Daijinkanboukouseikagakuka/0000080278.pdf]. In the context of the guideline, the investigators shall not necessarily be required to obtain informed consent; however, we made public information concerning this study on the web [http://www.fmu.ac.jp/univ/sangaku/data/koukai_2/2771.pdf] and provided opportunities for the research subjects to refuse the use of their personal information. Further, both ethics committee did not require individual informed consent from participants because all data were fully anonymized before all authors accessed them.

### Data collection

BP was measured using a standard sphygmomanometer or an automated measurement device on the right arm after a 5-min rest in a seated position during the first-year visit (2008 or 2009) and second-year visit (2009 or 2010). Therefore, BP levels on the first-year visit did not necessarily correspond to the BP level just before the oral administration of antihypertensive drugs, and the time of drug initiation between the first and second visits to the health check-up facilities could not be accurately determined.

Urine protein dipstick test results were recorded as (−), (±), (1+), (2+), and (3+). Blood sampling was performed in a fasting state. eGFR was calculated according to a revised equation for Japanese people stated below [21]:

$$eGFR\;\left(\frac{\frac{mL}{min}}{1.73\;m^{2}}\right)=194\times age\;{\left(years\right)}^{-0.287}\times serum\;creatinine\;{\left(\frac{mg}{dL}\right)}^{-1.094}(if\;female\times 0.739)$$

CKD was defined as eGFR <60 mL/min/1.73 m^2^ or urine protein dipstick test ≥1+. Participants answered a questionnaire concerning their medical history (cardiovascular disease, CVD), current medication (for hypertension, diabetes, and dyslipidemia), and lifestyle (smoking habit and alcohol drinking). CVD was defined as stroke (ischemic or hemorrhagic) and ischemic heart disease (angina and myocardial infarction). Diabetes was diagnosed based on the American Diabetes Association guidelines [22] as fasting plasma glucose ≥126 mg/dL, glycated hemoglobin ≥6.5%, or self-reported use of antidiabetic medications. Hypertension was defined as SBP ≥140 mmHg, BDP ≥90 mmHg, or self-reported use of antihypertensive medications. Dyslipidemia was defined according to the Japan Atherosclerosis Society guidelines [23] as LDL ≥140 mg/dL, HDL <40 mg/dL, TG ≥150 mg/dL, or self-reported use of anti-dyslipidemic medications.

### Statistical analysis

Statistical analyses were performed using SPSS version 20.0 J (IBM Corp., Armonk, NY). Data were expressed as mean ± SD, unless otherwise described. To investigate the association between baseline uric acid level and NOBP, participants were classified into quartiles based on uric acid levels. Logistic analysis was performed to determine the association between higher uric acid level and NOBP, with quartile 1 as a reference, and analysis was unadjusted and adjusted for age, sex, BMI, presence of diabetes, presence of CKD, presence of dyslipidemia, past CVD, daily drinking, and current smoking. First-visit BPs were not introduced because their values were not measured just before the use of antihypertensive drugs. Odds ratio (OR) for NOBP was similarly calculated by a 1 mg/dL increase in uric acid level. Adjusted ORs were further evaluated in the following subgroups: elderly vs. younger (65 years old), men vs. women, obese vs. non-obese (BMI, 25 kg/m^2^), diabetic vs. non-diabetic, CKD vs. non-CKD, and dyslipidemic vs. non-dyslipidemic.

## Results

### Participant characteristics

**Fig 2:**
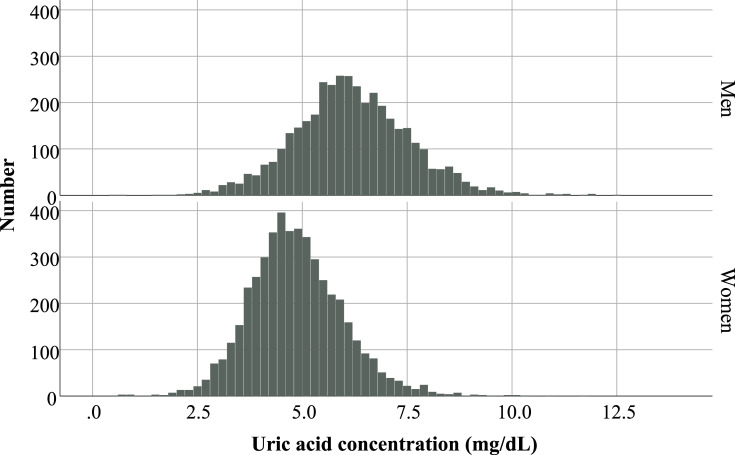
Histogram of baseline uric acid concentration by sex. Sex difference in uric acid concentration is prominent. In the histogram, the baseline uric acid concentration is distributed normally in both sexes. The mean (SD) values were 4.8 (1.1) mg/dL and 6.1 (1.4) mg/dL in women and men, respectively.

Participants were classified into quartiles according to their baseline uric acid level, and their characteristics are shown in Table 1. The mean age (standard deviation [SD]) was 65.5 (6.4) years, and 55.0% were women. The histogram of the baseline uric acid concentration by sex is normally distributed (Fig 2). The mean baseline uric acid level was 5.4 (1.4) mg/dL, which was 4.8 (1.1) mg/dL in women and 6.1 (1.4) mg/dL in men. First-visit SBP and DBP were 144 [20] mmHg and 83 [12] mmHg, respectively. The second-visit SBP and DBP were 134 [15] mmHg and 78 [10] mmHg, respectively. The prevalence rates of diabetes, CKD, dyslipidemia, obesity, past CVD, daily drinking, and current smoking were 13.3%, 23.8%, 57.7%, 32.1%, 10.6%, 26.7%, and 12.1%, respectively, of which sex difference was prominent among quartiles. Over 80% of quartile 1 was women. Similarly, the prevalence rates of CKD, obesity, daily drinking, and current smoking were different among quartiles; this implies a higher prevalence was observed in quartile 4. Throughout the selection process, several participants were excluded. Comparison between included and excluded participants is shown in S1 Table. First-visit BPs substantially differed between them; however, this is rational because all included participants were prescribed antihypertensive drug (s) a year after.

**Table 1:** Comparison between quartiles based on baseline uric acid concentration.

	Quartile 1	Quartile 2	Quartile 3	Quartile 4
Number	2245	2216	2188	2015
Uric acid, mg/dL	3.8 (0.6)	4.9 (0.3)	5.8 (0.3)	7.3 (0.9)
First-visit SBP	143 (19)	143 (20)	143 (19)	146 (20)
First-visit DBP	82 (12)	82 (12)	84 (12)	86 (13)
Second-visit SBP	133 (15)	133 (15)	134 (15)	135 (15)
Second-visit DBP	77 (10)	77 (10)	78 (10)	79 (10)
Age	66.0 (5.9)	65.9 (6.1)	65.5 (6.4)	64.5 (7.2)
Age ≥65 years old	66.9%	66.5%	64.5%	61.1%
Sex, women	82.9%	69.9%	43.7%	19.6%
FPG, mg/dL	100 (28)	99 (25)	100 (22)	102 (21)
HbA1c, %	5.89 (0.97)	5.84 (0.83)	5.81 (0.71)	5.80 (0.70)
Antidiabetic drug user	6.1%	5.8%	6.3%	4.5%
Diabetes mellitus	13.5%	13.0%	13.7%	13.0%
eGFR, mL/min/1.73 m^2^	78.1 (17.0)	75.1 (18.0)	73.4 (16.5)	69.1 (16.7)
Dipstick proteinuria, ≥ 1+	6.2%	7.1%	9.1%	14.0%
CKD	16.2%	19.2%	23.9%	37.1%
LDL, mg/dL	127 (30)	128 (32)	128 (32)	124 (32)
HDL, mg/dL	64.5 (15.8)	62.0 (15.3)	59.2 (15.2)	56.4 (16.6)
TG, mg/dL	112 (70)	121 (71)	135 (85)	167 (133)
Antidyslipidemic drug user	14.8%	13.9%	13.0%	9.4%
Dyslipidemia	52.7%	55.7%	60.8%	62.1%
BMI	22.8 (3.1)	23.5 (3.2)	24.2 (3.2)	24.7 (3.1)
Obesity, BMI ≥25 kg/m^2^	22.4%	28.5%	35.3%	43.3%
CVD history	9.0%	10.4%	11.3%	11.9%
Daily drinking	13.2%	19.1%	31.9%	45.1%
Current smoking	5.9%	9.5%	15.3%	18.5%

BMI, body mass index; CKD, chronic kidney disease; CVD, cardiovascular disease; DBP, diastolic blood pressure; SBP, systolic blood pressure; eGFR, estimated glomerular filtration rate; FPG, fasting plasma glucose; HbA1c, glycosylated hemoglobin; HDL, high-density lipoprotein; LDL, low-density lipoprotein; SBP, systolic blood pressure

TG, triglyceride.

### Logistic analysis

The rates of NOBP in quartiles 1–4 were 57.4%, 57.4%, 60.1%, and 66.7%, respectively. According to the optimal target BP categories, the NOBP rate of participants with diabetes or CKD, elderly or others, and non-elderly were 72.3%, 64.0%, and 37.2%, respectively (Table 2). Table 3 shows the OR for NOBP of baseline uric acid quartiles. In both unadjusted and adjusted models, Q4 was significantly associated with NOBP compared with Q1, the ORs and 95% CI (Q4 vs. Q1) of the unadjusted and adjusted values were 1.49 and 1.31–1.68 (p < 0.01) and 1.36 and 1.16–1.59 (p < 0.01), respectively. Table 4 shows the ORs for NOBP by a 1 mg/dL increase in baseline uric acid. Overall, the OR and 95% CI were 1.08 and 1.04–1.13 (p < 0.01), respectively. In subgroup analyses, the presence or absence of aging, obesity, and dyslipidemia did not affect the results. On the contrary, the association between higher uric acid level and NOBP was not significant in women, diabetic participants, and CKD participants. These associations were unchanged even if the first-visit SBP was added as a covariate following the performance of adjusted logistic analysis (S2 Table).

**Table 2:** Rate of non-attainment of optimal blood pressure (NOBP) level.

	NOBP Rate	*p*-value
Overall (n = 8,664)	60.2%	
Diabetes/CKD (n = 2,895)	72.3%	< 0.01
Elderly/others (n = 3,658)	64.0%	
Non-elderly (n = 2,111)	37.2%	

CKD, chronic kidney disease; Elderly, aged ≥ 65 years.

**Table 3:** Odds ratios for non-attainment of optimal blood pressure level by quartiles of baseline uric acid concentration.

	Unadjusted			Adjusted		
	OR	95% CI	p-value	OR	95% CI	p-value
Quartile 1	Reference			Reference		
Quartile 2	1.00	0.89–1.13	0.99	0.98	0.86–1.11	0.73
Quartile 3	1.12	0.99–1.26	0.07	1.02	0.89–1.18	0.74
Quartile 4	1.49	1.31–1.68	<0.01	1.36	1.16–1.59	<0.01

Adjusted by age, sex, body mass index, presence of diabetes/dyslipidemia/chronic kidney disease/past cardiovascular disease, daily drinking, and current smoking. OR, odds ratio; CI, confidence interval.

**Table 4:** Adjusted odds ratios for non-attainment of optimal blood pressure level of +1 mg/dL of uric acid concentration.

	OR	95% CI	p-value
Overall (n = 8,664)	1.08	1.04–1.13	<0.01
Age ≥65 years (n = 5,621)	1.07	1.02–1.13	0.01
Age <65 years (n = 3,043)	1.09	1.02–1.17	0.01
Men (n = 3,902)	1.10	1.04–1.16	<0.01
Women (n = 4,762)	1.06	0.998–1.13	0.06
BMI ≥25 kg/m^2^ (n = 2,782)	1.12	1.05–1.20	<0.01
BMI <25 kg/m^2^ (n = 5,882)	1.06	1.01–1.12	0.01
Diabetes (n = 1,155)	1.06	0.95–1.18	0.32
Non-diabetes (n = 7,509)	1.09	1.04–1.14	<0.01
CKD (n = 2,059)	1.05	0.97–1.14	0.22
Non-CKD (n = 6,605)	1.09	1.04–1.14	<0.01
Dyslipidemia (n = 4,999)	1.06	1.003–1.11	0.04
Non-dyslipidemia (n = 3,665)	1.12	1.05–1.20	<0.01

BMI, body mass index; CKD, chronic kidney disease; OR, odds ratio; CI, confidence interval.

## Discussion

We reported a significant association between higher baseline uric acid concentration and NOBP in a community-based cohort without severe kidney failure (eGFR <15 mL/min/1.73 m^2^). Recent basic studies clarified that higher uric acid concentration is involved in the activation of intrarenal renin-angiotensin system [12], suppression of endothelial nitric oxide production [10], and progression of arteriolar wall changes, such as narrowing [11] or hyalinosis [13]. These findings basically supported the presence of a significant association between hyperuricemia and prevalence of hypertension in cross-sectional studies [1–3] and incident hypertension in longitudinal studies [4–9]. Similarly, these findings indicated that higher uric acid concentration could be associated with resistance to hypertensive drug therapy, in other words, the NOBP level. This study equally demonstrated such relationship. Moreover, the incidence of hypertension and NOBP is quite a distinct issue; however, the underlying mechanism should be similar regarding the biological aspect. In addition, some social factors and the so called “clinical inertia” [24], could contribute to NOBP; the number of prescribed drugs may negatively affect the initiation of new drugs to prevent over-prescription even if NOBP is observed. Unfortunately, the database had no information on the number of antihypertensive drugs; therefore, this factor could not be accessed.

According to the subgroup analyses, age, BMI, and lipid level did not affect the relationship between uric acid concentration and NOBP; however, some conditions clearly affected this relationship, and the presence of diabetes or CKD negated this association. The aforementioned conditions should be strongly related to prevalent or incident hypertension via salt sensitivity, renin-angiotensin system activation, sympathetic nerve activation, endothelial damage, and volume retention, among others. [16, 17]. Some of these mechanisms are similar to those of hyperuricemia; however [10–13, 15, 16], those mechanisms should be strengthened in diabetic or CKD patients.

Another interesting result was the sex difference. The ORs for NOBP were higher in men than in women; however, the OR in women was almost different but not statistically significant. Recent studies concerning sex difference in the relationship between uric acid concentration and vascular damage showed conflicting results. Canepa et al. reported a significant relationship between longitudinal increase in uric acid concentration and longitudinal increase in pulse wave velocity (PWV) in the general population [25]. Similarly, this association was revealed to be prominent in men than in women. On the contrary, Nagano et al. reported that a longitudinal increase in PWV based on the baseline uric acid level was stronger in women than in men among normotensive population [26]. Further, Tanaka et al. investigated a hypertensive cohort to evaluate the influences of baseline uric acid level on vascular atherosclerotic markers [27]. In a cross-sectional study, higher uric acid level was significantly associated with lower flow-mediated dilation (FMD) in women, unlike men. A longitudinal study equally showed stronger association between higher uric acid level and lower FMD in women than in men. These inconsistent results on sex difference are occasionally observed in epidemiological studies; however, the causes were not usually obvious. These differences were attributed to the inclusion of different cohorts with or without comorbidities or different analytical methods used.

The contribution of a reduction in baseline uric acid concentration to the subsequent decline in BP remains uncertain. Feig et al. investigated young adults (mean age, 15 years, n = 30) with mild hypertension and hyperuricemia (uric acid >6 mg/dL) who were prescribed 200 mg of allopurinol for 4 weeks in a double-blind and cross-over design [28]. The casual SBP declined by 6.9 mmHg in the allopurinol group compared to -2.0 mmHg in the placebo group. It was concluded that allopurinol could reduce BP without antihypertensive drugs. However, the following factors should be considered: young age of participants, a mean BMI of 33 kg/m^2^, and a short test period. On the contrary, Ohta et al. reported that reducing baseline uric acid concentration did not contribute to BP control [29]. Their cohort was quite different from that of Feig et al. The mean age of their participants was 64 years and participants were almost receiving antihypertensive drugs. Only 20 participants were categorized into two arms: receiving febuxostat 40 mg or benzbromarone 50 mg, respectively, for 3 months in a cross-over design. BP remained unchanged before and after intervention. The small number of participants and the extreme age difference between these studies indicated the difficulty in determining the influence of uric acid-lowering treatment on BP.

This study is strengthened by its sufficient sample size which was adequate to classify participants into quartiles and analyze OR for NOBP. However, there were some limitations. First, the study period was approximately 1 year and an accurate timing of starting antihypertensive drug (s) or the classes and the number of drugs were unknown. However, observational period lasted for a year at the longest, and many antihypertensive drugs were not presumed to be commonly prescribed. Second, BP level just before starting the drug(s) was similarly unknown. Finally, many participants were excluded because of some missing laboratory data and information on antihypertensive drug (s) use.

In conclusion, this study obviously demonstrated that higher baseline uric acid level could have negative effects on antihypertensive therapy; therefore, hyperuricemia should be raised as one of the important factors in the management of hypertensive subjects.

## Supporting information

S1 Table Comparison between included and excluded participants. (DOCX)

S2 Table Adjusted odds ratios for non-attainment of optimal blood pressure level by quartiles of baseline uric acid concentration. (DOCX)
