# Supplementary material for: Higher baseline uric acid concentration is associated with non-attainment of optimal blood pressure
Source: PLoS One. 2020 Jul 27;15(7):e0236602. doi: 10.1371/journal.pone.0236602 (PMC7384644; doi:10.1371/journal.pone.0236602)
Supplement: S1 Table — (DOCX) [file pone.0236602.s001.docx]

**S1 Table. Comparison between included and excluded participants**

|  | Included | Excluded |
| --- | --- | --- |
| Number | 8664 | 452,354 |
| Uric acid, mg/dL | 5.4 (1.4) | 5.2 (1.4), n=334,730 |
| First-visit SBP | 144 (20) | 129 (17), n=452,162 |
| First-visit DBP | 83 (12) | 76 (11), n=452,144 |
| Second-visit SBP | 134 (15) | 128 (17), n=280,491 |
| Second-visit DBP | 78 (10) | 75 (10), n=280,471 |
| Antihypertensive drug user | 0% | 29.8%, n=452,228 |
| Age | 65.5 (6.4) | 63.4 (8.2), n=418,456 |
| Age ≥65 years old | 64.9% | 55.1%, n=418,438 |
| Sex, women | 55.0% | 58.9%, n=452,354 |
| FPG, mg/dL | 100 (24) | 98 (21), n=310,640 |
| Ab A1c, % | 5.84 (0.81) | 5.76 (0.71), n=428,182 |
| Antidiabetic drug user | 5.7% | 5.1%, n=452,219 |
| eGFR, mL/min/1.73m^2^ | 74.1 (17.4) | 75.2 (16.8), n=387,494 |
| Dipstick proteinuria, ≥1+ | 9.0% | 5.4%, n=451,347 |
| CKD | 23.8% | 19.4%, n=389,768 |
| LDL, mg/dL | 127 (31) | 125 (31), n=452,249 |
| HDL, mg/dL | 60.6 (16.0) | 61.5 (16.1), n=452,305 |
| TG, mg/dL | 133 (94) | 128 (84), n=452,311 |
| Anti-dyslipidemic drug user | 12.9% | 15.0%, n=452,222 |
| BMI | 23.8 (3.2) | 23.3 (3.4), n=452,297 |
| CVD history | 10.6% | 9.1%, n=422,846 |
| Daily drinking | 26.7% | 23.0%, n=429,002 |
| Current smoking | 12.1% | 14.4%, n=452,221 |

BMI, body mass index; CKD, chronic kidney disease; CVD, cardiovascular disease; DBP, diastolic blood pressure; eGFR, estimated glomerular filtration rate; FPG, fasting plasma glucose; HbA1c, glycosylated hemoglobin; HDL, high-density lipoprotein; LDL, low-density lipoprotein; SBP, systolic blood pressure; TG, triglyceride
