# Supplementary material for: Higher baseline uric acid concentration is associated with non-attainment of optimal blood pressure
Source: PLoS One. 2020 Jul 27;15(7):e0236602. doi: 10.1371/journal.pone.0236602 (PMC7384644; doi:10.1371/journal.pone.0236602)
Supplement: S2 Table — (DOCX) [file pone.0236602.s002.docx]

**S2 Table. Adjusted odds ratios for non-attainment of optimal blood pressure level by quartiles of baseline uric acid concentration**

|  | OR | 95% CI | p-value |
| --- | --- | --- | --- |
| Quartile 1 | Reference | |  |
| Quartile 2 | 0.99 | 0.86-1.13 | 0.85 |
| Quartile 3 | 1.05 | 0.91-1.21 | 0.49 |
| Quartile 4 | 1.34 | 1.14-1.58 | <0.01 |

Adjusted by age, sex, body mass index, first-visit systolic blood pressure, presence of diabetes/dyslipidemia/chronic kidney disease/past cardiovascular disease, daily drinking, and current smoking. OR, odds ratio; CI, confidence interval.
